# Tim17 and Stress Adaptation in *Trypanosoma brucei*

**DOI:** 10.3390/pathogens15101053

**Published:** 2026-10-04

**Authors:** Minu Chaudhuri, Railyn Webster, Hira Karim

**Affiliations:** Department of Microbiology, Immunology, and Physiology, School of Medicine, Meharry Medical College, Nashville, TN 37208, USA; railyn.webster@mmc.edu (R.W.); hira.karim@mmc.edu (H.K.)

**Keywords:** mitochondrial, stress response, *Trypanosoma brucei*, Tim17, ROS, SLS response

## Abstract

*Trypanosoma brucei*, a parasitic protozoan, is the infectious agent of a deadly disease in humans and livestock known as African trypanosomiasis. TbTim17 is the major component of the TbTIM complex that imports hundreds of nuclear-encoded proteins into the mitochondria. Here, we show that depletion of TbTim17 increased *T. brucei* tolerance to paraquat, increasing the EC50 by 1.6- to 1.9-fold compared with the wild type. Subsequent analysis revealed that increased reactive oxygen species levels resulting from TbTim17 knockdown preadapt cells to resist paraquat-induced oxidative stress. This is supported by the finding that treating cells with N-acetylcysteine during TbTim17 RNAi induction reduced the paraquat EC50 to wild-type levels. TbTim17 knockdown reduced the levels of several mitochondrial proteins, except SOD A. These cells were also tolerant to heat stress. Interestingly, in wild-type parasites, TbTim17 levels increased under oxidative and heat stress, suggesting it has a protective role under stress. Furthermore, TbTim17 knockdown induces the SLS response, a trypanosome-specific ER stress-response pathway, suggesting a communication between two organelles during stress in *T. brucei*. Understanding the role of mitochondrial protein translocases in the mitochondrial stress response in *T. brucei* is critical for elucidating the mechanisms of adaptation to environmental stresses and drug resistance in this parasite.

## 1. Introduction

*Trypanosoma brucei*, a divergent eukaryotic parasite, is the causative agent of a fatal disease known as African sleeping sickness in humans and Nagana in ruminants, posing a major health concern and hindering economic development in the affected areas [1]. *T. brucei* possesses a single reticular mitochondrion per cell that not only plays a vital role in parasite survival but also modulates its structure and metabolic pattern for adapting under different environmental conditions during parasite differentiation [2]. However, mitochondrial stress response pathways are not clearly defined in this organism. Furthermore, trypanosomatids mostly lack transcriptional control of gene expression [3]; thus, retrograde signaling from mitochondria to the nucleus to activate the canonical transcription factors is not found.

As in other eukaryotes, most mitochondrial proteins in *T. brucei* are nucleus-encoded and imported into the mitochondrion from the cytosol [4,5]. *T. brucei* translocates proteins and tRNAs into the mitochondrion via the atypical ATOM and the TbTIM17 complexes of the organellar outer and inner membranes, respectively [6,7]. The TbTIM17 is a mega-Dalton-sized protein complex, consisting of a few relatively conserved proteins and several trypanosome-specific components [6,8]. TbTim17 is the major component of the complex and belongs to the Tim17/22/23 family of proteins [9]. In other eukaryotes, Tim17 and Tim23 form the TIM23 complex, whereas Tim22 forms the TIM22 complex [10,11]. In contrast to the three proteins in this family in other eukaryotes, *T. brucei* possesses only TbTim17. The Tim17/22/23 family proteins (17–23 kDa) have four transmembrane domains centrally located, leaving both the N- and C-terminal hydrophilic regions in the intermembrane space [4,10,11]. Yeast has one copy of each, but multiple copies of these proteins are found in plants and mammals. Humans have two copies of Tim17 (Tim17A and Tim17B) [12,13]. Tim17B is constitutively expressed, whereas Tim17A expression varies across tissues and is upregulated in many forms of cancer [14]. It has been shown that Tim17A in humans and Tim23 in *Caenorhabditis elegans* play crucial roles in the mitochondrial unfolded protein response (UPRmt) [15,16]. Mitochondrial stress triggers degradation of Tim17A by the mitochondrial protease YME1L, perturbing mitochondrial import of the transcription factor ATFS-1, which under normal conditions is imported into the mitochondria and quickly degraded by the matrix protease LONP. ATFS-1 possesses a mitochondrial targeting signal (MTS) and a nuclear localization signal (NLS). Thus, unimported ATFS-1 accumulates in the nucleus and activates expression of various chaperones and mitochondrial biogenesis proteins to alleviate the stress [15,16]. The mitochondrial stress response is also connected to the integrated stress response pathways (ISR), which involve many endoplasmic reticulum (ER) and cytosolic proteins that lead to translational attenuation via phosphorylation of the translation initiation factor eIF2-α [17]. Besides, there are several other mitochondrial quality control (MQC) pathways. These include mitochondrial precursor overaccumulation stress (mPOS) [18], the constitutively active mitochondrial protein translocation-associated degradation (mitoTAD) [19], and others. In these cases, translocation-impaired proteins are ubiquitinated in the cytosol and degraded by the proteasome.

Stress response pathways have also been studied in trypanosomes. A mostly investigated one is the ER-stress response by splice-leader silencing (SLS-response) [20]. Inhibition of protein translocation in the ER or accumulation of unfolded proteins in the ER lumen triggers down-regulation of the synthesis of SL-RNA, which is a piece of RNA that needs to be trans-spliced to the 5′-end of each mRNA to make it functional in trypanosomes [3]. Thus, SL-RNA silencing reduced the ER protein load and induced apoptotic-like cell death upon consistent stress. Although well-established in the procyclic form that dwells in the insect gut, the existence of the SLS response and an associated upregulation of the ER-chaperone protein, BiP, is controversial in the mammalian infective bloodstream forms of *T. brucei* [21]. It is also not clear if a global translation inhibition by eIF2 phosphorylation exists in trypanosomes. Therefore, the ISR pathway is still very sketchy in this parasite. Another study on the MQC pathway showed that a nuclear-localized ubiquitin-like protein (TbUbl1) is released into the cytosol and is required for the degradation of accumulated precursor proteins when mitochondrial protein import is impaired [22]. It has also been shown that nutritional stress causes an accumulation of tRNAThr halves in mitochondria, which associate with mitochondrial ribosomes and stimulate translation, thereby increasing *T. brucei* survival [22,23].

Modulation of mitochondrial activities is critical for *T. brucei* differentiation during its digenetic life cycle. In the mammalian stage, the bloodstream form of *T. brucei* survives by aerobic glycolysis, utilizing glucose as its primary energy source [24]. This form lacks cytochrome-dependent respiratory complexes but possesses trypanosome alternative oxidase (TAO) [25]. Whereas, upon differentiation to the procyclic form in the insect gut, where glucose is scarce, *T. brucei* utilizes amino acids as its major energy source. At this stage, the mitochondrion is fully developed, with cytochrome-dependent OXPHOS complexes, and TAO levels are reduced. Thus, this form has two terminal oxidases in mitochondria, and the flow of electrons can be flexible. Electron transfer via TAO is non-proton-motive and is not coupled to ATP synthase. It acts as an electron overflow mechanism, thereby reducing mitochondrial oxidative stress [26,27].

Here, we show that *T. brucei* possesses a ROS-mediated signaling mechanism to attenuate mitochondrial stress. Forced downregulation of TbTim17 stimulates this defense mechanism. Furthermore, TbTim17 levels were moderately upregulated under stress, suggesting it has a protective role in mitochondria. Persistent oxidative stress induced by TbTim17 depletion activates the SLS response, which likely leads to cell death.

## 2. Materials and Methods

Cell lines, growth medium, and cell growth analysis: The procyclic form of *T. brucei* 427 29-13 expressing T7 polymerase and tetracycline repressor genes was grown in SDM-79 medium containing 10% heat-inactivated fetal bovine serum in the presence of hygromycin (50 μg/mL), G418 (15 μg/mL) [28] and was used as the parental control for our experiments. *T. brucei* cells expressing TbTim17 RNAi (TbTim17 KD) and TbTim62 RNAi (TbTim62 KD) were previously developed [29,30]. These cell lines were maintained in the same medium supplemented with phleomycin (2.5 μg/mL). Cell growth was assessed by inoculating the procyclic form at a cell density of 2 × 10^6^/mL in fresh medium. Cells were counted at different growth time points with a Neubauer hemocytometer. The log of the cumulative cell number was plotted against incubation times.

Subcellular fractionation and crude mitochondria isolation: *T. brucei* mid-log phase cells (2 × 10^8^) were resuspended in 500 μL of SMEP buffer (250 mM sucrose, 20 mM morpholinepropanesulfonic acid [MOPS]-KOH, pH 7.4, 2 mM EDTA, 1 mM phenylmethylsulfonyl fluoride [PMSF]) containing 0.03% digitonin and incubated on ice for 5 min. The cell suspension was then centrifuged for 5 min at 6800× *g* at 4 °C. The resultant pellet was considered the crude mitochondrial fraction, and the supernatant contained soluble cytosolic proteins.

Paraquat treatment and EC50 determination: For determination of the growth inhibitory concentration (EC50) of paraquat, mid-log phase procyclic form cells were inoculated in fresh medium as described above in 24-well plates. Calculated amounts of paraquat (Sigma-Aldrich, St. Louise, MO, USA) (stock of 20 mM in H_2_O) were added to the culture to reach the final concentrations of 0 to 200 μM. Triplicate wells were set up for each concentration. Cells were counted each day for 2 days in each well, twice. Standard deviations for the growth inhibition curve were calculated from four independent experiments. EC50 was determined from normalized non-linear regression plots of percent inhibition of cell growth versus log of paraquat concentrations using GraphPad Prism (version 10). Standard errors were calculated from 3 independent datasets.

Measurement of mitochondrial membrane potential: Cells were harvested and suspended in fresh culture medium at a density of 4–5 × 10^6^/mL. MitoTracker^®^ Red CMXROS (Molecular Probe^®^, Waltham, MA, USA) was dissolved in DMSO at a concentration of 1 mM and added to the cell suspension at a final concentration of 0.5 μM. The mixtures were incubated at 27 °C for 10 min. Cells were then washed and incubated in fresh culture medium for an additional 30 min. Cells were washed twice with 1 X PBS and fixed in 0.37% paraformaldehyde at 4 °C for 5 min. Next, cells were centrifuged, washed and resuspended in cold PBS and stored at 4 °C until FACS analysis. Fluorescence intensity (Geo. Mean) was measured with a FACSCalibar (Becton Dickinson, Franklin Lakes, NJ, USA) analytical flow cytometer using absorption at 578 nm and emission at 599 nm.

Measurement of cellular ROS: *T. brucei* cells (1 × 10^7^/mL) were incubated with 10 μM 2′,-7′-dichlorodihydrofluorescein diacetate (DCFH-DA) (ThermoFisher, Waltham, MA, USA) for 30 min at 27 °C. After incubation, cells were washed once with 1 X PBS and analyzed by flow cytometry by measuring the fluorescence emission at 520 nm using an excitation wavelength of 492 nm.

Puromycin incorporation assay: Log-phase cells were incubated in the presence and absence of puromycin (Sigma-Aldrich, St. Louise, MO, USA) (2 μg/mL) in complete medium for 1 h. Cells were harvested by centrifugation, washed 3 times with 1 X PBS, and lysed with SDS-PAGE sample buffer. Total cellular proteins were analyzed by SDS-PAGE and immunoblot analyses using anti-puromycin antibody as the probe.

SDS-PAGE and Immunoblot analysis: Proteins from isolated mitochondria and total cell extracts were resolved on a 12% SDS/PAGE gel and then transferred to nitrocellulose membranes (Bio-Rad, Hercules, CA, USA). The antigens were visualized using a West Pico Chemiluminescent Substrate (Thermo Fisher, Waltham, MA, USA). Antibodies against TbTim17 [29], TbTim62 [30], SOD A [31], TAO [32], mHsp70 [33], VDAC [34], and TbBiP [35] were used as probes. Anti-puromycin antibody was purchased (Cell Signaling, Danvers, MA, USA). The secondary antibodies used were either anti-rabbit or anti-mouse immunoglobulins linked to horseradish peroxidase (Thermo Fisher, Waltham, MA, USA).

**qRT-PCR:** For quantitative real-time PCR (qRT-PCR) analysis, RNA was isolated from *T. brucei* procyclic cells using Trizol reagent (Thermo Fisher, Waltham, MA, USA) according to the manufacturer’s protocol. RNA was treated with RNase-Free Turbo DNase (Thermo Fisher, Waltham, MA, USA) at 37 °C for 30 min, and RNA was recovered by phenol/chloroform extraction and ethanol precipitation. The cDNA was made from 1.0 μg of RNA(DNase-digested) using the iScript cDNA synthesis kit (Bio-Rad, Hercules, CA, USA). Real-time quantitative PCR was performed with a CFX96 Touch real-time PCR detection system (Bio-Rad, Hercules, CA, USA). The resulting cDNA was amplified using SsoAdvanced universal SYBR green supermix (Bio-Rad, Hercules, CA, USA) and gene specific primers targeting the following transcripts: spliced leader RNA (nt 26 to 108), 7SL RNA, gene ID Tb927.8.2861 (nt 147 to 221), TbSNAP42, gene ID Tb927.5.3910 (nt 703 to 811), and actin A, gene ID Tb927.9.8850 (nt 1031 to 1091), as described [21]. Spliced leader RNA transcripts were normalized to 7SL RNA, while TbSNAP42 transcript levels were normalized to actin levels. All reactions were performed in technical triplicate, and standard deviations were calculated from three biological replicates.

**Densitometry and statistical analyses:** ImageJ software (version 1.53a, NIH) was used to perform densitometry of Western blots. The band intensity of the loading control (Ponceau-stained band around 50 kDa region) was used for normalization, and percent control values were plotted using MS Excel (Version 16.113.3). Standard deviations were calculated from three independent experiments for each blot. Statistical significance was calculated by *t*-test in Excel. Statistical significance of some experiments was also calculated by one-way ANOVA in GraphPad Prism (Version 11) as indicated.

## 3. Results

### 3.1. TbTim17 Depletion Increased T. brucei Tolerance to Paraquat

TbTim17 is the major component of the single TIM complex in *T. brucei* and is essential for the import of hundreds of nuclear-encoded proteins destined for the mitochondrial matrix and inner membrane. To investigate whether TbTim17 depletion elicits a stress response, we induced TbTim17 RNAi in procyclic cells with doxycycline for 48 h. After that, we treated cells with different concentrations of paraquat, which induces oxidative stress, and monitored cell growth for the next 48 h. We used parental (29-13) and TbTim62 RNAi procyclic cells in parallel to compare them. TbTim62 is also a component of the TbTIM17 complex, but its abundance is lower, and the effect of its depletion on mitochondrial protein import is less than that of TbTim17 [6].

**Figure 1:**
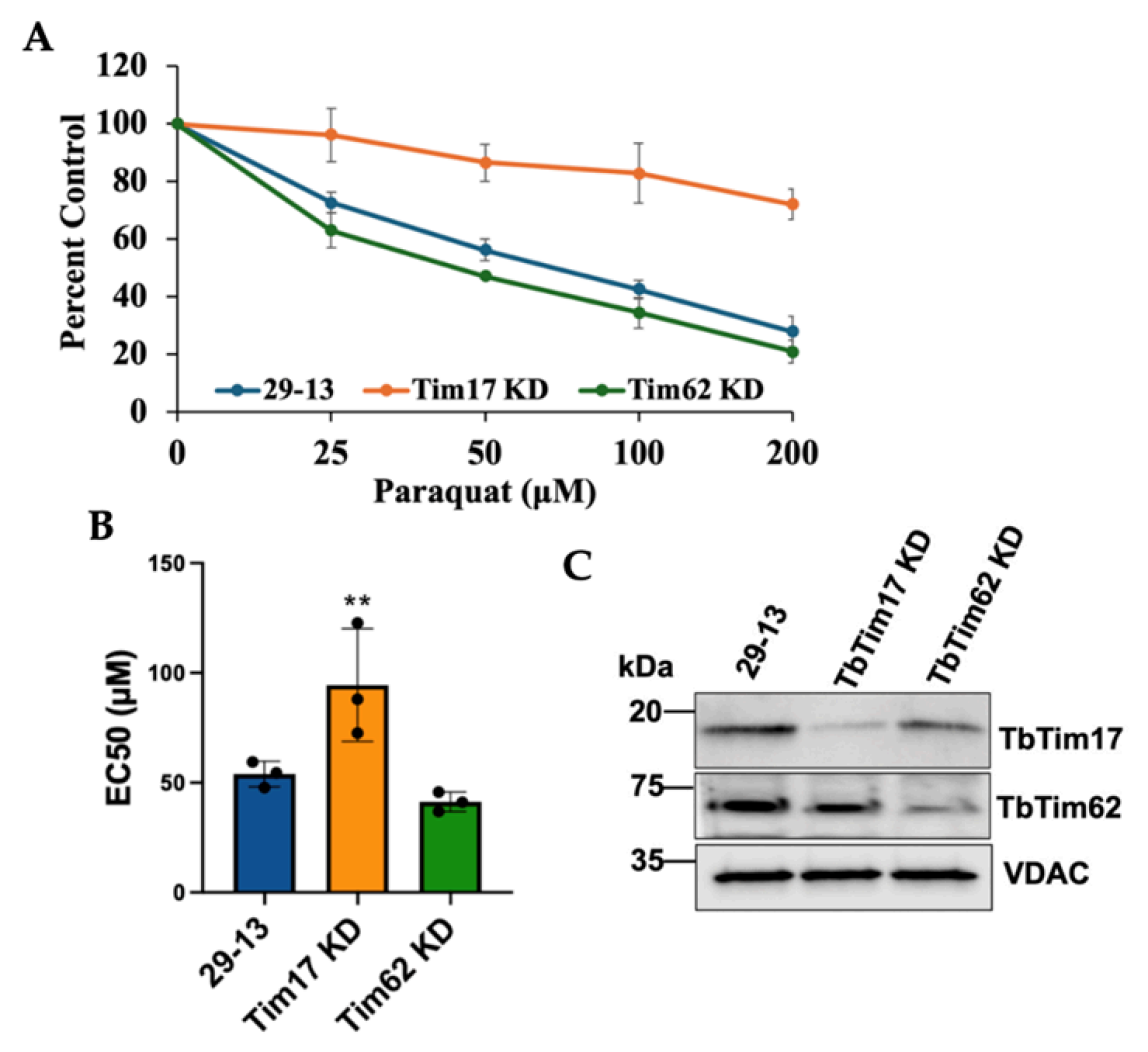
**TbTim17 knockdown in *T. brucei* increased the EC50 of paraquat.** The TbTim17 RNAi and TbTim62 RNAi cell lines were induced with doxycycline for 2 days to knockdown (KD) the target proteins. These cells and the parental control *T. brucei* 29-13 were then treated with different concentrations of paraquat (0–200 μM), and cell growth was monitored for 48 h. (**A**). Percentage of cell number relative to the untreated control was plotted against different concentrations of paraquat. Standard deviations (SDs) were calculated from 4 independent experiments. (**B**). EC50 values of paraquat for 29-13, TbTim17 KD, and TbTim62 KD cells were calculated as described in the Materials and Methods and plotted using GraphPad Prism. Significance values were calculated by one-way ANOVA and are indicated by asterisks (**, *p* < 0.05). (**C**). Immunoblot analysis of equal amounts of mitochondrial proteins isolated after 48 h of induction of RNAi was performed using antibodies for TbTim17 and TbTim62. *T. brucei* 29-13 cells were grown for the same time and used in parallel. VDAC was used as the loading control.

We found that TbTim17-depleted cells are more resistant to paraquat in comparison to the 29-13 and TbTim62-depleted procyclic cells (Figure 1A). The EC50 values were determined from three independent experiments by non-linear regression plots of the percent inhibition of cell growth versus log of paraquat concentrations (Appendix A.1). Results showed that the EC50 of paraquat for 29-13 was 53.9 ± 5.7 μM, whereas that for TbTim17 knockdown cells was 93.91 ± 16.7 μM. Thus, TbTim17 knockdown cells have 1.6- to 1.9-fold higher EC50 for paraquat in comparison to 29-13 (Figure 1B, Appendix A.1). TbTim62 knockdown cells behave similarly to 29-13 and have an EC50 of 41.28 ± 5.22 μM. These results indicated that TbTim17 depletion equipped *T. brucei* cells to resist paraquat-induced oxidative stress. In other eukaryotes, reduced mitochondrial protein import elicits a stress response to maintain cell homeostasis. Therefore, our results suggest TbTim17 depletion may induce a similar stress response phenomenon in *T. brucei*. Immunoblot analysis of the mitochondrial proteins isolated from cells after 48 h of RNAi induction showed depletion of TbTim17 and TbTim62 levels in respective cell lines, as expected (Figure 1C).

### 3.2. Increased ROS Due to TbTim17 Depletion Promotes Stress Tolerance

It has been shown in mammals and certain nematodes that mitochondrial stress response occurs via retrograde signaling by ATF transcription factors [15,16]. However, there are few studies on mitochondrial stress response in *T. brucei*. In particular, *T. brucei* lacks transcriptional control of gene expression and any ATF homolog.

**Figure 2:**
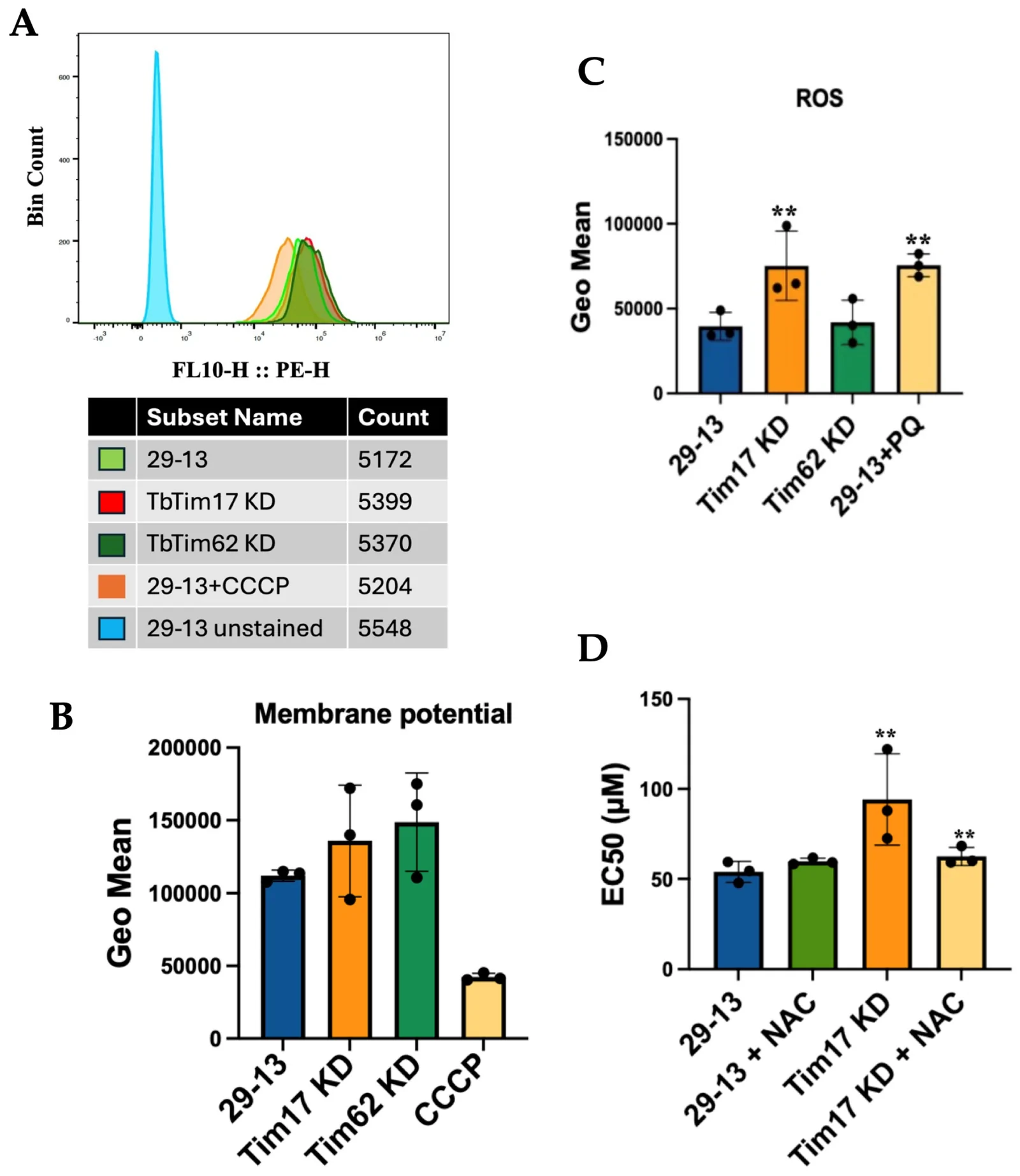
**Measurement of mitochondrial membrane potential and ROS levels and treatment of cells with NAC.** (**A**). *T. brucei* 29-13, TbTim17 RNAi, and TbTim62 RNAi cells induced with doxycycline for 48 h were stained with MitoTracker Red, fixed with 0.37% paraformaldehyde, and fluorescence intensity was measured at 599 nm by fluorescence-activated cell sorter (FACS) analysis. The representative data are shown. Wild-type cells treated with 50 μM CCCP served as a control to demonstrate mitochondrial membrane potential depletion. More than 5000 cells were counted for each sample. (**B**). The geo mean values from three independent experiments were plotted for different cell types. (**C**). *T. brucei* 29-13, TbTim17 RNAi, and TbTim62 RNAi cells induced with doxycycline for 48 h were stained with DCFH-DA, and fluorescence intensity was measured by FACS analysis. Fluorescence intensities (Geo mean) were plotted for different cell types. Experiments were done in triplicate. (**D**). The TbTim17 RNAi cells were induced with doxycycline for 48 h in the absence and presence of NAC (100 μM). *T. brucei* 29-13 cells were also treated with NAC for the same time. EC50 for paraquat (PQ) was determined as described above and plotted. SDs were calculated from 3 independent experiments. Significance values were calculated by one-way ANOVA and are indicated by asterisks (**, *p <* 0.05).

To investigate why TbTim17-depleted *T. brucei* is more tolerant to paraquat, we assessed stress markers, such as altered mitochondrial membrane potential and ROS, at the early stages (within 48 h) of TbTim17 RNAi induction. Mitochondrial membrane potential was measured by MitoTracker staining followed by flow-cytometric analysis. A slight elevation of the membrane potential was observed in the RNAi cells; however, the changes were not significant in comparison to the parental control (Figure 2A and B). Mitochondrial membrane potential in 29-13 cells was depleted when treated with CCCP, as expected. Next, we assessed the levels of ROS in TbTim17-depleted and control cells using DCFH-DA (Figure 2C). We noticed that the levels of ROS were significantly higher in TbTim17-depleted cells in comparison to 29-13 and TbTim62-depleted *T. brucei*. The 29-13 cells treated with paraquat were used as the positive control. We observed that paraquat treatment increased ROS levels in 29-13 cells, as expected. Therefore, we conclude that TbTim17 knockdown specifically increased the levels of ROS.

Increased cellular ROS is detrimental to cellular function; however, a certain level of ROS often generates a stress signal that mitigates its effects [36]. Therefore, to investigate whether the increased ROS levels in TbTim17 knockdown cells are the cause of these cells becoming more tolerant to paraquat, we treated cells with N-acetyl cysteine (NAC), an antioxidant, during RNAi induction and determined the EC50. The 29-13 cells pretreated with NAC did not show any significant changes in the EC50 of paraquat. However, TbTim17 knockdown cells pretreated with NAC showed a significant reduction in EC50 compared with those without NAC pretreatment (Figure 2D). These results show that increased ROS in TbTim17-depleted cells induces a stress response, possibly by altering the levels of certain stress-tolerant proteins.

### 3.3. TbTim17 Depletion Increases SODA Levels

**Figure 3:**
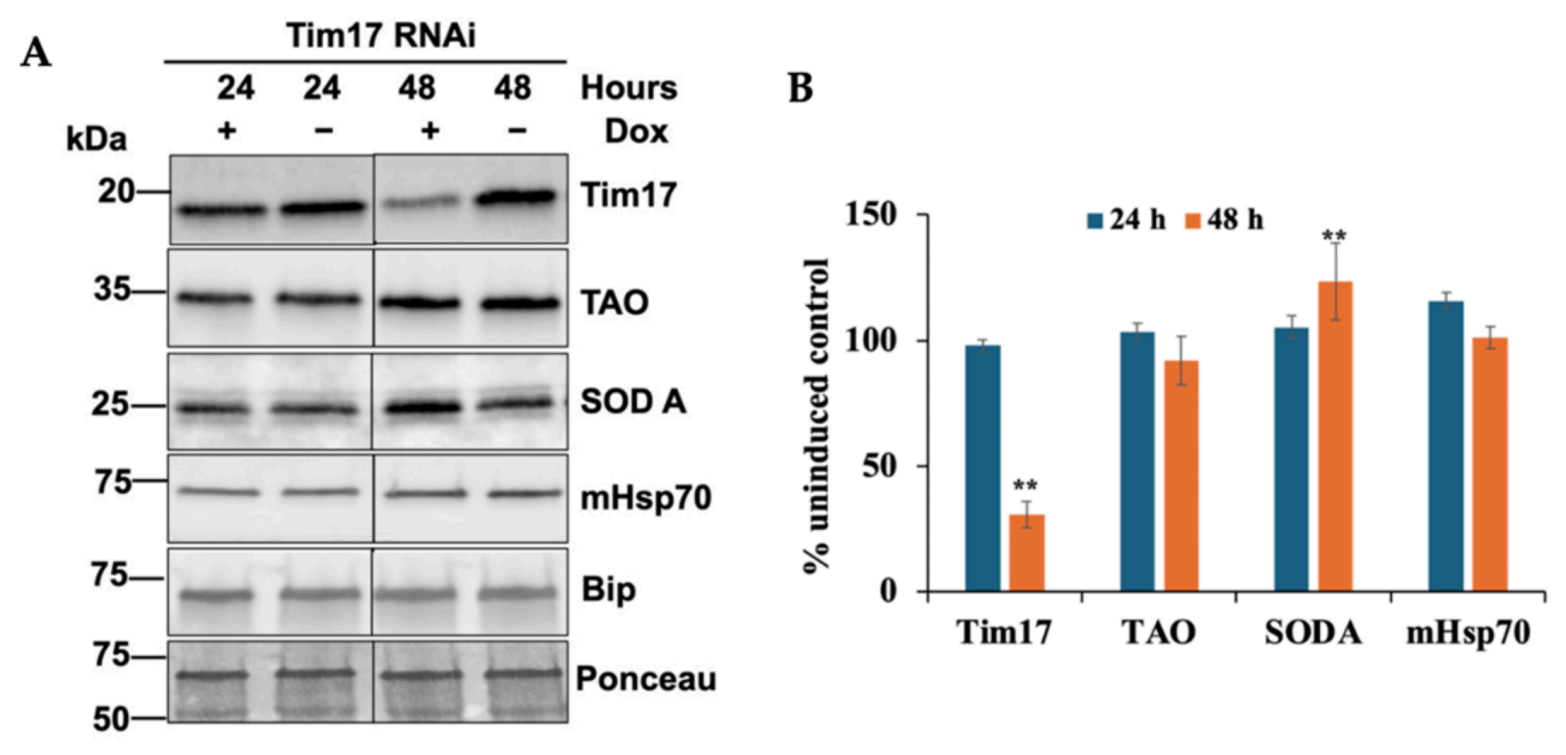
**Effect of TbTim17 knockdown on the chaperones and antioxidant proteins in *T. brucei*.** (**A**). Immunoblot analysis of proteins in the crude mitochondrial fractions isolated from TbTim17 RNAi cells grown in the presence (+) and absence (−) of doxycycline for 24 and 48 h. Blots were probed with the indicated antibodies. Ponceau-stained blot (50 kDa region) is shown as a loading control. (**B**). Quantitation of the immunoblot results. Band intensities were quantified in ImageJ, normalized to the corresponding Ponceau-stained bands, and plotted as a percent of the respective uninduced control. SDs were calculated from three independent experiments. Statistical significances were calculated by Student’s *t*-test and is indicated by asterisks (**, *p <* 0.05).

We wanted to investigate if TbTim17 depletion induces the expression of chaperones like mitochondrial Hsp70 as well as oxidative stress response proteins such as mitochondrial SOD A and TAO. Results from the immunoblot analysis show that TbTim17 levels decreased by about 80% within 48 h of RNAi induction (Figure 3A). It was expected that the levels of mitochondrial proteins would be reduced due to inhibition of their import into mitochondria in TbTim17 knockdown cells. Although we observed a downregulation of TAO and mHsp70, as expected, the levels of SOD A increased. This is possibly due to increased ROS levels in these cells. Together, this suggests that increased ROS due to TbTim17 knockdown acted as a signaling factor, upregulating SOD A; thus, these cells became more resistant to paraquat-induced oxidative stress.

### 3.4. TbTim17 Level Is Upregulated Under Oxidative Stress

**Figure 4:**
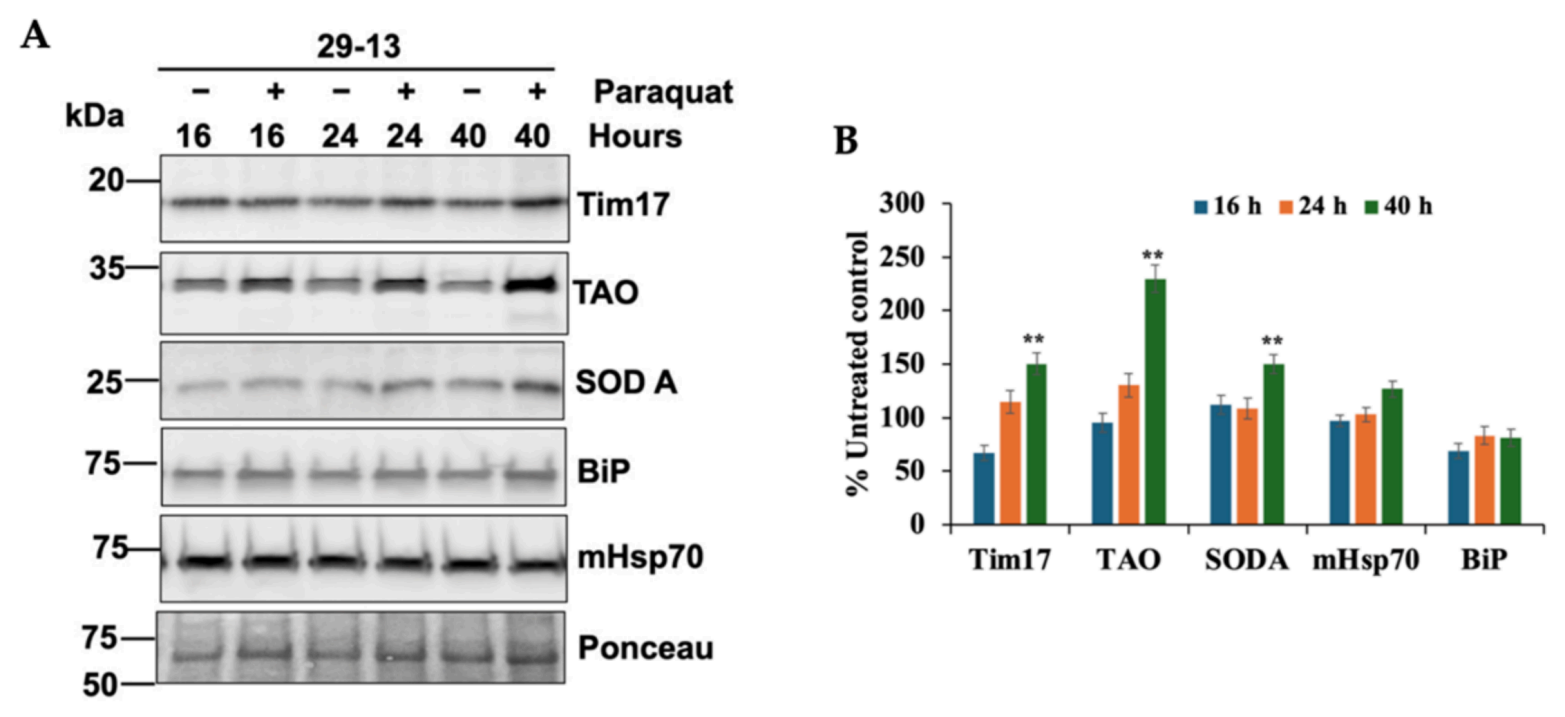
**Effect of paraquat treatment on the chaperones and antioxidant proteins in *T. brucei*.** (**A**). Immunoblot analysis of proteins in the crude mitochondrial fractions isolated from *T. brucei* 29-13 cells treated (+) and kept untreated (−) for different time points (16, 24, and 40 h). Blots were probed with the indicated antibodies. Ponceau-stained blot (50 kDa region) is shown as a loading control. (**B**). Quantitation of the immunoblot results. Band intensities were quantified in ImageJ, normalized to the corresponding Ponceau-stained bands, and plotted as a percent of untreated control. SDs were calculated from three independent experiments. Statistical significances were calculated by Student’s *t*-test and is indicated by asterisks (**, *p <* 0.05).

Previous reports in other eukaryotes demonstrated that oxidative stress reduced Tim17 levels by its degradation via the mitochondrial protease YME1L to induce oxidative stress response [15]. Therefore, we sought to determine whether oxidative stress alters TbTim17 levels in *T. brucei*. To induce oxidative stress, we treated 29-13 cells with paraquat (100 μM) for 16 to 40 h and assessed levels of TbTim17, TAO, SOD A, mHsp70, and BiP. We found that, upon paraquat treatment, TbTim17 levels increased over time, as did TAO and SOD A (Figure 4A,B). Paraquat generates ROS via its redox cycling [37]. Thus, to reduce cellular ROS, TAO may pass excess electrons directly to oxygen to form water, and its level was upregulated upon paraquat treatment. In contrast, mHsp70 and BiP were not significantly upregulated. We also exposed these cells for a shorter time (0–6 h) and assessed the protein levels. We observed no significant changes in the levels of TbTim17, TAO, SODA, mHsp70, and BiP (Appendix A.2). Together, these findings suggest that TbTim17 levels increased along with antioxidant proteins under stress, possibly to protect mitochondrial function by importing more proteins.

### 3.5. TbTim17 Knockdown Cells Are More Tolerant to Heat Stress

**Figure 5:**
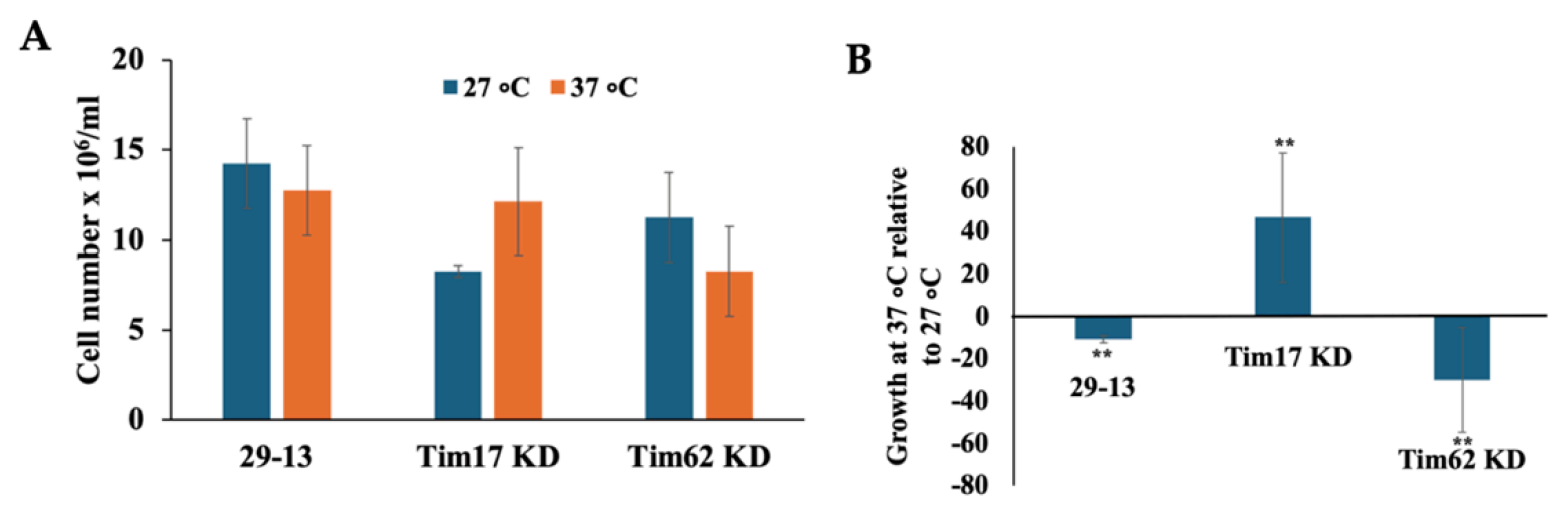
**Heat tolerance of the parental and TbTim17 and TbTim62 knockdown *T. brucei*.** TbTim17 RNAi and TbTim62 RNAi cells were induced with doxycycline for 48 h and further incubated at different temperatures (27 °C and 37 °C) for 16 h. The parental 29-13 cells were treated similarly for the same period. (**A**). Cell numbers were calculated and plotted for each type of cell as indicated. SDs were calculated from three independent experiments. (**B**). Cell growth at 37 °C relative to that at 27 °C for the respective cell types was calculated in percent and plotted. Statistical significances were calculated by a *t*-test and is indicated by asterisks (**, *p <* 0.05).

*T. brucei* encounters a quick shift in temperature from 27 °C to 37 °C during its life cycle [1,2]. Thus, to investigate whether TbTim17 plays a role in heat stress, we compared cell growth of the control and TbTim17 knockdown *T. brucei* at normal (27 °C) and at a higher temperature (37 °C). After induction of TbTim17 RNAi for 2 days, we split the culture and incubated it separately for 16 h at 27 °C and 37 °C. The 29-13 and TbTim62 RNAi cells were used as controls in parallel. Interestingly, we found that TbTim17 knockdown cells are more tolerant to heat stress than the 29-13 and TbTim62 RNAi cells (Figure 5A,B). We observed a 10–20% growth retardation of 29-13 cells due to incubation at 37 °C for 16 h (Figure 5A). In contrast, TbTim17 knockdown cells grew better at 37 °C than at 27 °C. Following TbTim17 knockdown, *T. brucei* cell growth was reduced to 50% compared to the control at 27 °C. However, after shifting to 37 °C, these cells grew similarly to control cells at 37 °C (Figure 5A,B). Instead, Tim62 knockdown reduced 15–20% cell growth in comparison to control at 27 °C, and it is reduced more when transferred at 37 °C (Figure 5A,B).

These results show that TbTim17 knockdown cells are better equipped to withstand the stress induced by a temperature increase from 27 °C to 37 °C. It is well known that heat stress denatures proteins, forms protein aggregates, increases ROS levels, accelerates protein denaturation and aggregation and inhibits cell growth [38]. It can be speculated that TbTim17 knockdown cells have higher levels of antioxidant proteins, such as SOD A. Therefore, these cells, when transferred at higher temperatures, can mitigate ROS levels that favor cell growth.

### 3.6. TbTim17 Level Is Upregulated During Heat Stress

**Figure 6:**
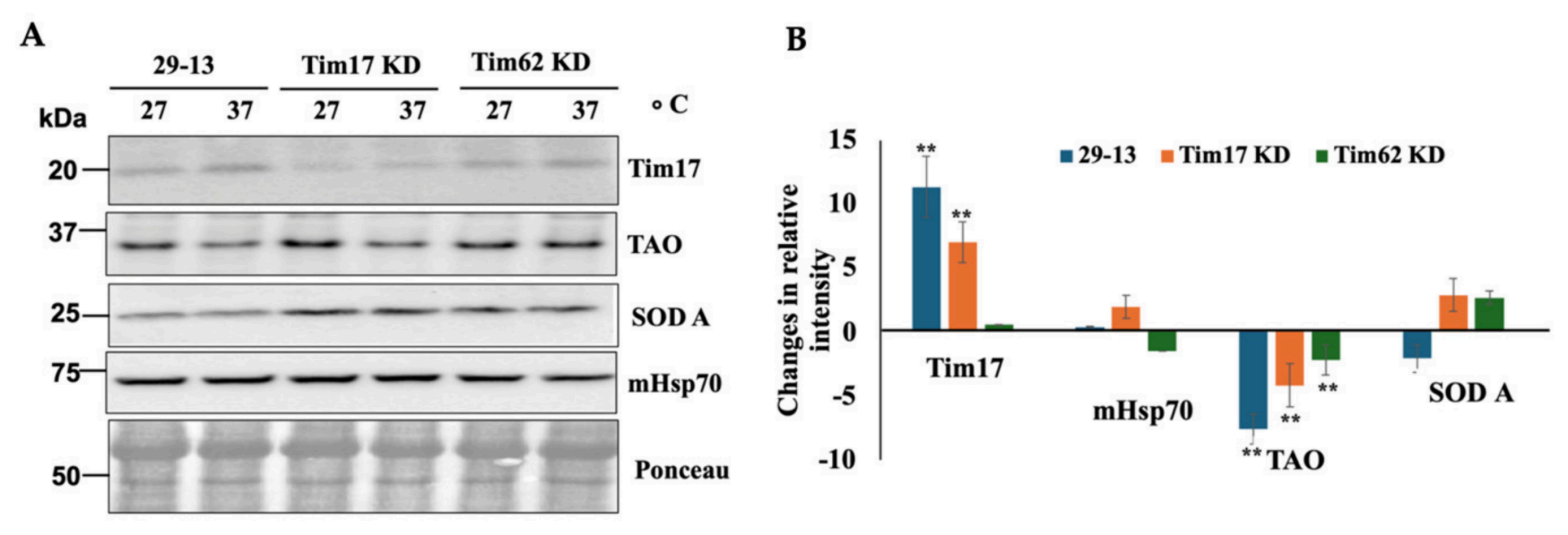
**Effect of heat stress on mitochondrial protein levels.** (**A**). TbTim17 RNAi and TbTim62 RNAi cells were induced with doxycycline for 48 h and further incubated at different temperatures (27 °C and 37 °C) for 16 h. The parental 29-13 cells were treated similarly for the same period. Cells were harvested, and total cellular proteins were analyzed by immunoblotting using the indicated antibodies. (**B**). Band intensities were quantified by ImageJ software, normalized with the corresponding Ponceau-stained bands. Changes in the intensity of each protein band at 37 °C relative to that at 27 °C in each cell type were calculated as percentages and plotted. SDs were calculated from three independent experiments. Statistical significances were calculated by Student’s *t*-test and is indicated by asterisks (**, *p <* 0.05).

To investigate how heat stress alters the levels of mitochondrial proteins, we compared the levels of Tim17, TAO, SOD A, and mHsp70 in 29-13, TbTim17 RNAi, and TbTim62 RNAi cells incubated at 27 °C and 37 °C. Surprisingly, we observed that TbTim17 levels were consistently higher in 29-13 cells grown at 37 °C than in those grown at 27 °C (Figure 6A,B). We observed that TAO levels were reduced due to heat stress across all three cell types. Since TAO is thermogenic, reduced respiration via TAO could be a compensatory mechanism at higher temperatures. However, we could not exclude other possibilities, such as a decrease in protein stability. SOD A levels were upregulated upon heat treatment in 29-13 cells, as expected. This is also aligned with the previous report [39]. However, mHsp70 levels were minimally affected.

### 3.7. TbTim17 Knockdown Induces SLS Response

**Figure 7:**
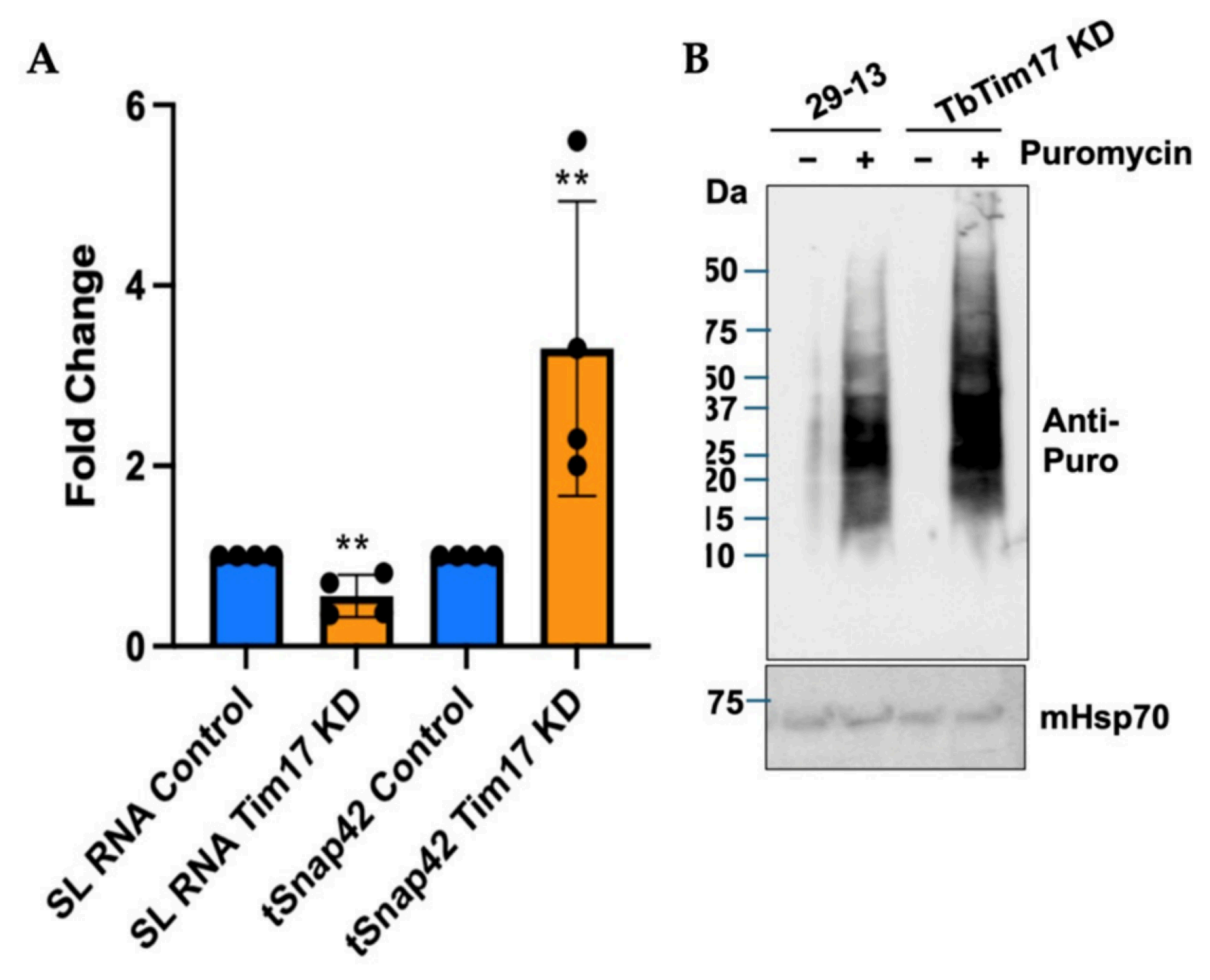
**Effect of TbTim17 RNAi on the levels of SL-RNA and tSNAP42 transcripts.** (**A**). qRT-PCR analyses were performed to estimate the levels of SL-RNA and tSNAP42 transcripts. Total RNA was isolated from *T. brucei* 29-13 and TbTim17 RNAi cells induced with doxycycline for 48 h. cDNA was synthesized from DNase-treated RNA, and qRT-PCR was performed as described in the Materials and Methods. SL RNA levels were normalized to 7SL, and tSNAP42 transcript levels were normalized to actin. Results from four independent biological replicates are presented. Significance values were calculated by one-way ANOVA and are indicated by asterisks (**, *p <* 0.05). (**B**). Assay for puromycin incorporation in newly synthesized proteins. *T. brucei* 29-13 and TbTim17 RNAi cells induced with doxycycline for 48 h were incubated with puromycin (2 μg/mL) for 1 h. Cells were washed, and total cellular proteins were analyzed by SDS-PAGE and immunoblot analysis probed with anti-puromycin antibody.

One specific stress response phenomenon in *T. brucei* is the SLS response, which is activated upon ER stress. During this response, the SL RNA transcription is blocked because tSNAP42, a specific transcription factor for SL RNA synthesis, fails to bind to the SL RNA promoter. Consequently, tSNAP42 accumulates in the nucleus [40]. To examine if TbTim17 RNAi could induce the SLS response, we quantified the transcript levels of SL RNA and tSNAP42 in 29-13 and TbTim17 knockdown *T. brucei* by qRT-PCR. We found a ~50% reduction in SL-RNA and 2 to 5-fold increase in the level of tSNAP42 transcript in TbTim17 knockdown cells in comparison to the control (Figure 7A). These results indicate that inhibition of mitochondrial protein import by TbTim17 knockdown induces the SLS response to reduce the protein load in the mitochondria.

To examine if TbTim17 depletion induces a global translation inhibition, we performed a puromycin incorporation assay. Puromycin acts as an analog of tyrosyl tRNA, enters the ribosome’s A-site, covalently binds to the growing peptide chain, and causes premature termination. Therefore, puromycin incorporation into protein can be used to measure active translation in cells [41]. From the incubation of the 29-13 and TbTim17 knockdown cells in the presence of puromycin and analysis of the total cellular proteins by immunoblot using anti-puromycin antibody, we found that incorporation of puromycin in the newly synthesized proteins are very similar to both types of cells, showing that stresses induced by TbTim17 RNAi for 48 h did not induce a global translation inhibition (Figure 7B). These results were not unexpected as it has been shown that *T. brucei* does not respond to stress by translational inhibition mediated by eIF2 phosphorylation like in other eukaryotes [42]. Instead, SLS response induces apoptotic-like cell death in *T. brucei* when the stress persists [20]. Therefore, TbTim17 knockdown initiates a ROS-mediated signaling event that induces an SLS response and likely proceeds to apoptotic-like cell death.

## 4. Discussion

Here, we demonstrated that TbTim17, a major and essential component of the mitochondrial protein import machinery, plays a role in the mitochondrial stress response; however, it acts differently from its counterparts in other eukaryotes. Depletion of TbTim17 increased ROS levels within 24–48 h, which could be due to a reduction in the import of multiple nuclear-encoded mitochondrial proteins simultaneously or to the inhibition of the import of specific labile protein(s) needed to maintain ROS levels. Consequently, mitochondrial Fe-SOD A levels were upregulated. SOD A converts ROS to hydrogen peroxide. Trypanosomes lack catalase but possess a strong peroxidase system, including tryparedoxin peroxidase (TXN) and glutaredoxin 1 (GrX1) [43], which neutralize peroxides via a glutathione/trypanothione redox transfer chain to maintain cell homeostasis. SOD A is a nuclear-encoded protein that is required to be imported into the mitochondrial matrix via the translocase system. Therefore, upregulation of SOD A protein levels in TbTim17-depleted cells suggests that this protein may be stabilized under these conditions, possibly via post-translational modification. However, other mechanisms for its upregulation cannot be excluded. We demonstrated that the increased ROS levels are responsible for the oxidative stress tolerance of TbTim17-depleted cells. This phenomenon fits well with the increased SOD A levels. Previous studies showed that SOD A transcript levels are upregulated in the G1 and G2 phases of the cell cycle [44], and SOD A plays a role in *T. brucei* life-cycle adaptation and virulence [45,46]. Therefore, the parasite must have specific mechanisms to regulate it.

TAO expression is also very flexible. TAO is significantly more abundant in the bloodstream forms than in the procyclic form [25]. It is upregulated in the metacyclic form and is modulated under certain experimental and environmental changes [2], suggesting involvement in adaptive or regulatory functions pertinent to survival and life-cycle progression. TAO bypasses electron flow and reduces ROS levels during perturbations of electron flow through the cytochrome-dependent respiratory pathway [25]. We observed an upregulation of TAO in the 29-13 procyclic cells treated with paraquat. This further showed the importance of TAO in cellular adaptation under oxidative stress.

TbTim17 is the single homolog of Tim17/22/23 family proteins in trypanosomes. Native gel electrophoresis revealed that TbTim17 forms multiple-sized protein complexes by associating with several other trypanosome-specific Tim proteins, like TbTim62 [6,47]. A previous report showed that TbTim62 knockdown reduced the abundance of the TbTIM17 complex and the steady-state levels of TbTim17 [30]. However, TbTim62 depletion did not increase ROS levels, unlike TbTim17 RNAi within 2 days. This indicates that TbTim17 RNAi reduces the levels of TbTim17, the direct target of the RNAi, below a certain threshold at this time point, which is not reached by the secondary effect of TbTim62 RNAi.

In addition to oxidative stress, TbTim17-depleted *T. brucei* was more adaptable to a change in temperature from 27 °C to 37 °C. It is speculated that this tolerance is also due to elevated SOD A levels in TbTim17 RNAi cells. However, we could not eliminate the existence of other mechanisms. Interestingly, we found that TbTim17 levels were upregulated in the 29-13 procyclic cells under both heat and oxidative stresses. This may be a response to upregulate mitochondrial proteomes under stress, when mitochondrial proteins are damaged by oxidation and heat stress. Tim17 is known to protect mitochondrial DNA in other eukaryotes [48], although the mechanism underlying this function remains unknown. Whether TbTim17 has a similar function in *T. brucei* is worth investigating. During heat stress, we observed downregulation of TAO in all *T. brucei* cell lines tested, which is also very interesting and likely correlated with TAO’s thermogenic property or its stability.

Organellar communication is critical to maintain cellular homeostasis. In this regard, communication between the ER and mitochondria is well established. In *T. brucei*, perturbation of ER protein translocation induces an SLS response by activating the ER-associated kinase, PK3, which, in turn, phosphorylates the TATA-binding protein, TRF4, in the nucleus, thereby perturbing tSNAP42 binding to the SL-RNA promoter site, silencing SL-RNA transcription, and causing tSNAP42 to accumulate in the nucleus [49]. It has recently been shown that depletion of TbRHOM1, a TbTim17-associated protein, induces an SLS response to a lesser extent than does depletion of Sec63, the ER translocase [50]. This shows that stresses induced by mitochondrial protein import inhibition are transmitted to the ER. This report also showed that depletion of Golgi-localized quiescin sulfhydryl oxidase (QSOX) and ER oxidoreduction by RNAi induces the SLS response [50]. Here, we further showed that the depletion of TbTim17 reduces the levels of SL-RNA transcripts by about 50% compared with the control. TbTim17 reduction also increased the levels of tSNAP42 transcript by about 4- to 5-fold. This indicates that stresses induced by TbTim17 depletion in mitochondria are transmitted to the ER and induce the SLS response. Apparently, this SL RNA silencing had no significant effect on overall protein translation. This is consistent with the previous observation that the SLS response induces an apoptosis-like phenomenon in the procyclic form of *T. brucei* to eliminate unfit cells from the population [20].

Altogether, our results showed that TbTim17 plays a protective role in mitochondria. Heat or oxidative stress consistently increased TbTim17 levels, likely replenishing mitochondrial proteomes. TbTim17 depletion induces an ROS-mediated defense mechanism to mitigate stress caused by mitochondrial protein import inhibition, as we found that these cells were more tolerant to both heat and oxidative stresses. However, consistent depletion of TbTim17 is detrimental; it induces the SLS response that likely leads to apoptotic cell death. Therefore, TbTim17 is not only essential but also a stress-adaptive protein; thus, TbTim17 may warrant further investigation as a potential therapeutic target.

## Figures and Tables

**Figure A1:**
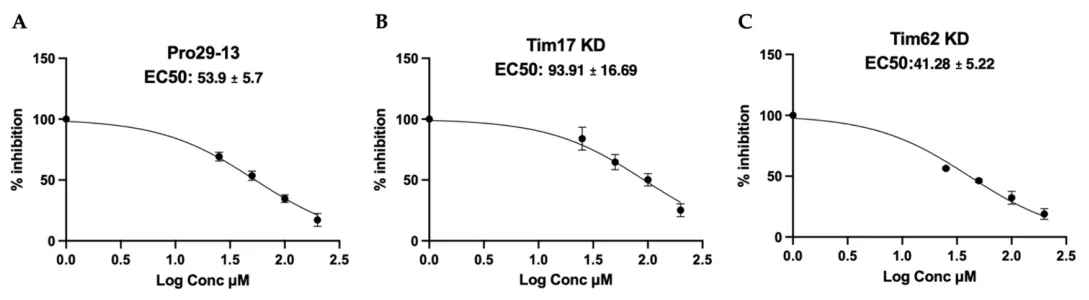
(**A**–**C**) EC50 determination of paraquat. EC50 values of paraquat for 29-13 (**A**), Tim17 KD (**B**), and Tim62 KD (**C**) were determined from normalized non-linear regression plots of percent inhibition of cell growth versus log of paraquat concentrations using GraphPad Prism (version 11). The standard errors were calculated in triplicate.

**Figure A2:**
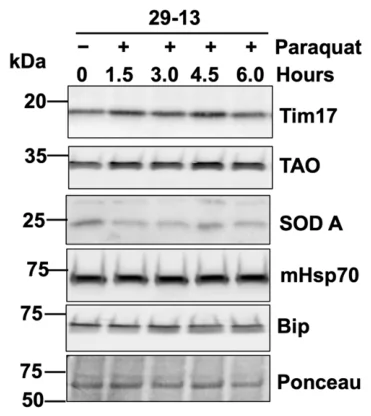
Effect of paraquat treatment at earlier time points on the chaperones and antioxidant proteins in *T. brucei.* Immunoblot analysis of proteins in crude membrane fractions isolated from *T. brucei* 29-13 cells treated (+) and kept untreated (−) at different time points (0–6 h). Blots were probed with the indicated antibodies. Ponceau-stained blot (50 kDa region) is shown as a loading control.

## Data Availability

The original contributions presented in this study are included in the article. Further inquiries can be directed to the corresponding author.

## Abbreviations

The following abbreviations are used in this manuscript:

ATFS-1	Activating transcription factor associated with stress-1
ATOM	Archaic translocase of mitochondrial outer membrane
BiP	Binding immunoglobulin protein
DCFH-DA	2′,-7′-dichlorodihydroFfuorescein diacetate (DCFH-DA)
DMSO	Dimethyl sulfoxide
EC50	Half maximal effective concentration
eIF2	Eukaryotic translation initiation factor
GrX1	Glutaredoxin-1
ISR	Integrated stress response
LONP	Lon peptidase (LON: *lon* mutant gene in bacteria)
mHSP70	Mitochondrial heat shock protein 70
mitoTAD	Mitochondrial protein translocation associated degradation
mPOS	Mitochondrial precursor overaccumulation stress
MTS	Mitochondrial targeting signal
MQC	Mitochondrial quality control
NAC	N-acetyl cysteine
NLS	Nuclear localization signal
OXPHOS	Oxidative phosphorylation
QSOX	Quiescin sulfhydryl oxidase
RNAi	RNA interference
ROS	Reactive oxygen species
SL-RNA	Splice Leader RNA
SLS	Splice leader silencing
SOD A	Superoxide dismutase A
TAO	Trypanosome alternative oxidase
TbTim17	*T. brucei* translocase of mitochondrial inner membrane 17
TbUb11	*T. brucei* ubiquitin-like 1
TbRHOM1	*T. brucei* rhomboid 1
TIM23	Translocase of mitochondrial inner membrane 23
TIM22	Translocase of mitochondrial inner membrane 22
tSNAP42	Trypanosomal small nuclear RNA activating protein 42
TXN	Tryparedoxin peroxidase
UPRmt	Unfolded protein response in mitochondria
VDAC	Voltage-dependent anion channel
YME1L	Yeast mitochondrial escape 1 like
